# Echinacoside Inhibits Glutamate Release by Suppressing Voltage-Dependent Ca^2+^ Entry and Protein Kinase C in Rat Cerebrocortical Nerve Terminals

**DOI:** 10.3390/ijms17071006

**Published:** 2016-06-24

**Authors:** Cheng Wei Lu, Tzu Yu Lin, Shu Kuei Huang, Su Jane Wang

**Affiliations:** 1 Department of Anesthesiology, Far-Eastern Memorial Hospital, Pan-Chiao District, New Taipei City 22060, Taiwan; drluchengwei@gmail.com (C.W.L.); drlin1971@gmail.com (T.Y.L.); nskh9450n@yahoo.com.tw (S.K.H.); 2 Department of Mechanical Engineering, Yuan Ze University, Taoyuan 32003, Taiwan; 3 School of Medicine, Fu Jen Catholic University, No. 510, Zhongzheng Rd., Xinzhuang Dist., New Taipei 24205, Taiwan

**Keywords:** echinacoside, glutamate release, cerebrocortical nerve terminals, voltage-dependent Ca^2+^ channels, protein kinase C

## Abstract

The glutamatergic system may be involved in the effects of neuroprotectant therapies. Echinacoside, a phenylethanoid glycoside extracted from the medicinal Chinese herb *Herba* *Cistanche*, has neuroprotective effects. This study investigated the effects of echinacoside on 4-aminopyridine-evoked glutamate release in rat cerebrocortical nerve terminals (synaptosomes). Echinacoside inhibited Ca^2+^-dependent, but not Ca^2+^-independent, 4-aminopyridine-evoked glutamate release in a concentration-dependent manner. Echinacoside also reduced the 4-aminopyridine-evoked increase in cytoplasmic free Ca^2+^ concentration but did not alter the synaptosomal membrane potential. The inhibitory effect of echinacoside on 4-aminopyridine-evoked glutamate release was prevented by ω-conotoxin MVIIC, a wide-spectrum blocker of Cav2.2 (N-type) and Cav2.1 (P/Q-type) channels, but was insensitive to the intracellular Ca^2+^ release-inhibitors dantrolene and 7-chloro-5-(2-chloropheny)-1,5-dihydro-4,1-benzothiazepin-2(3H)-one (CGP37157). Furthermore, echinacoside decreased the 4-aminopyridine-induced phosphorylation of protein kinase C, and protein kinase C inhibitors abolished the effect of echinacoside on glutamate release. According to these results, we suggest that the inhibitory effect of echinacoside on evoked glutamate release is associated with reduced voltage-dependent Ca^2+^ entry and subsequent suppression of protein kinase C activity.

## 1. Introduction

Echinacoside is a major phenylenthanoid glycoside present in *Herba* *Cistanche,* a well-known traditional Chinese medicine used to treat forgetfulness, impotency, and chronic constipation [1]. Echinacoside possesses various bioactivities such as antioxidation, anti-inflammation, anticancer, hepatoprotection, and immune modulation [2,3,4]. Notably, echinacoside has neuroprotective effects; for example, it can protect against oxidative stress- or neurotoxin-induced neurotoxicity in primary rat cortical neurons, human neuroblastoma SH-SY5Y cells, and pheochromocytoma (PC12) cells [5,6,7,8]. Furthermore, echinacoside attenuates brain damage and improves cognitive function in animal models of Parkinson’s disease, Alzhermer’s disease, and middle cerebral artery occlusion [9,10,11,12]. However, the mechanism through which echinacoside induces neuroprotection is not fully understood.

Neuroprotection is a complex process of preserving neuronal structure and function upon toxic insults. Glutamate excitotoxicity reduction is considered a potential mechanism involved in brain neuroprotection. Glutamate, an excitatory amino acid neurotransmitter, has a crucial role in several brain functions [13]. However, overactivation of glutamate receptors under high glutamate concentrations causes intracellular Ca^2+^ overload, mitochondrial dysfunction, free radical production, and neuronal death [14,15]. This pathological process is implicated in numerous brain disorders including cerebral ischemia, traumatic brain injury, epilepsy, and neurodegenerative disease [16,17]. Hence, inhibitors blocking pathophysiological glutamatergic transmission are considered a potential neuroprotective drugs. Notable examples of these are glutamate receptor antagonists [18,19]; however, clinical trials for these drugs have failed because of less effectivity and undesired, or even cytotoxic side effects [20,21]. In addition to direct glutamate receptor blockade, glutamate release inhibition may be an effective strategy for neuroprotection. Several neuroprotectants (e.g., memantine and riluzole) can reduce glutamate release in rat brain tissues [22,23,24].

Considering the role of glutamate in excitotoxicity and the neuroprotective profile of echinacoside, the present study used isolated nerve terminals (synaptosomes) purified from the rat cerebral cortex to investigate the effect of echinacoside on glutamate release and further explored potential mechanisms. The isolated nerve terminal preparation is a well-established model for studying the presynaptic regulation of neurotransmitter release by drugs in the absence of any postsynaptic effects [25]. By using this model, we evaluated the effect of echinacoside on glutamate release, membrane potential, presynaptic Ca^2+^ influx, and protein kinase C activity. According to our review of the literature, this is the first report documenting the mechanism through which echinacoside inhibits endogenous glutamate release at the presynaptic level.

## 2. Results

### 2.1. Echinacoside Inhibits 4-Aminopyridine-Evoked Glutamate Release from Rat Cerebrocortical Nerve Terminals by Reducing Vesicular Exocytosis

**Figure 1:**
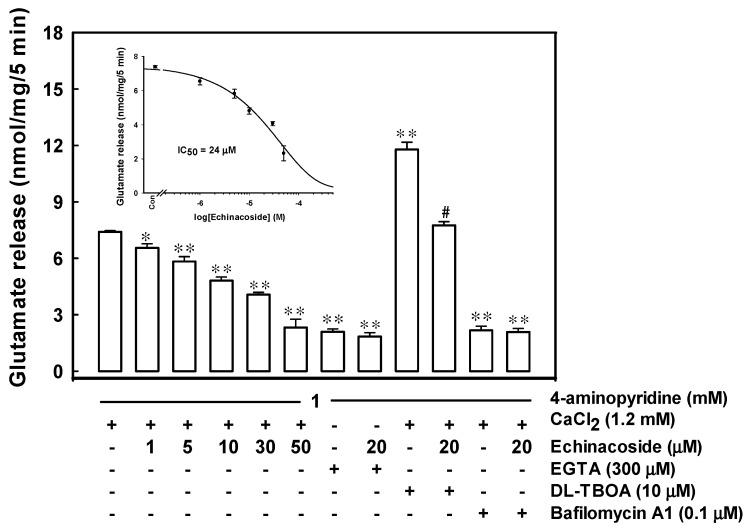
Echinacoside inhibits 4-aminopyridine-evoked glutamate release from rat cerebrocortical nerve terminals via the Ca^2+^-dependent exocytotic component. Glutamate release was evoked by 1 mM 4-aminopyridine in the absence (control) or presence of echinacoside (1, 5, 10, 30, and 50 μM), 300 μM ethylene glycol bis(β-aminoethyl ether)-*N*,*N*,*N*′,*N*′-tetraacetic acid (EGTA) (omitting CaCl_2_), 300 μM EGTA (omitting CaCl_2_) and 20 μM echinacoside, 10 μM dl-threo-beta-benzyl-oxyaspartate (dl-TBOA), 10 μM dl-TBOA and 20 μM echinacoside, 0.1 μM bafilomycin A1, or 0.1 μM bafilomycin A1 and 20 μM echinacoside. Echinacoside was added 10 min before depolarization and, EGTA, dl-TBOA or bafilomycin A1, 20 min prior to this. Results are mean ± S.E.M. of 4–6 independent synaptosomal preparations. *, *p* < 0.01, **, *p* < 0.001 versus control group. #, *p* < 0.05 versus the dl-TBOA-treated group.

Figure 1 illustrates the concentration-dependent effect of echinacoside on 4-aminopyridine-evoked glutamate release from purified rat cerebrocortical synaptosomes. In synaptosomes incubated with 1 mM CaCl_2_, 1 mM 4-aminopyridine evoked a glutamate release of 7.4 ± 0.1 nmol/mg/5 min, which was reduced by 1, 5, 10, 30, and 50 µM echinacoside to 6.5 ± 0.2, 5.8 ± 0.3, 4.8 ± 0.2, 4.1 ± 0.1, or 2.3 ± 0.4 nmol/mg/5 min, respectively (F(5,24) = 67.1, *p* = 0.000). The IC_50_ value for echinacoside-mediated inhibition of 4-aminopyridine-evoked glutamate release, derived from a dose-response curve, was 24 µM. Moreover, the glutamate release evoked by 1 mM 4-aminopyridine in an extracellular Ca^2+^-free solution containing 300 µM ethylene glycol bis(β-aminoethyl ether)-*N*,*N*,*N*′,*N*′-tetraacetic acid (EGTA) was 2.1 ± 0.2 nmol/mg/5 min (F(2,12) = 310.65, *p* = 0.000), and this Ca^2+^-independent component of 4-aminopyridine-evoked glutamate release was unaffected by 20 µM echinacoside (1.8 ± 0.2 nmol/mg/5 min; *p* = 0.58; Figure 1). In synaptosomes treated with 0.1 µM bafilomycin A1, a vesicular transporter inhibitor [26], 4-aminopyridine-evoked glutamate release was reduced significantly (2.2 ± 0.2 nmol/mg/5 min; F (2,12) = 249.518, *p* = 0.000). In the presence of bafilomycin A1, 20 µM echinacoside failed to significantly inhibit the release of glutamate (2.1 ± 0.2 nmol/mg/5 min; *p* = 0.94; Figure 1). By contrast, 10 µM dl-threo-beta-benzyl-oxyaspartate (dl-TBOA, a glutamate reuptake inhibitor) [27], increased 4-aminopyridine-evoked glutamate release to 11.8 ± 0.4 nmol/mg/5 min (*t*(8) = −11.31, *p* = 0.000). Even in the presence of dl-TBOA, 20 µM echinacoside inhibited 4-aminopyridine-evoked glutamate release significantly (7.7 ± 0.2 nmol/mg/5 min; F(2,12) = 87.23, *p* = 0.000; Figure 1).

### 2.2. Echinacoside Reduces Cytosolic Ca^2+^ Concentration but Does Not Alter the Synaptosomal Membrane Potential

Synaptosome depolarization caused by 1 mM 4-aminopyridine increased Ca^2+^ concentration (*p* = 0.000; Table 1). The application of 20 µM echinacoside did not significantly affect basal Ca^2+^ concentration (*t*(8) = 0.06, *p* = 0.95) but significantly reduced the 4-aminopyridine-induced increase in Ca^2+^ concentration (*t*(10) = 6.16, *p* = 0.000). In addition, 1 mM 4-aminopyridine increased in 3’,3’,3’-dipropylthiadicarbocyanine iodide [DiSC_3_(5)] fluorescence (*p* = 0.000). The addition of 20 µM echinacoside did not alter the resting membrane potential (t(8) = 0.976, *p* = 0.36) or significantly change the 4-aminopyridine-mediated increase in DiSC_3_(5) fluorescence (*t*(8) = −0.014, *p* = 0.99; Table 1).

### 2.3. Reduced Ca^2+^ Influx through the Cav2.2 (N-Type) and Cav2.1 (P/Q-Type) Channels May Be Associated with the Inhibition of 4-Aminopyridine-Evoked Glutamate Release by Echinacoside

**Figure 2:**
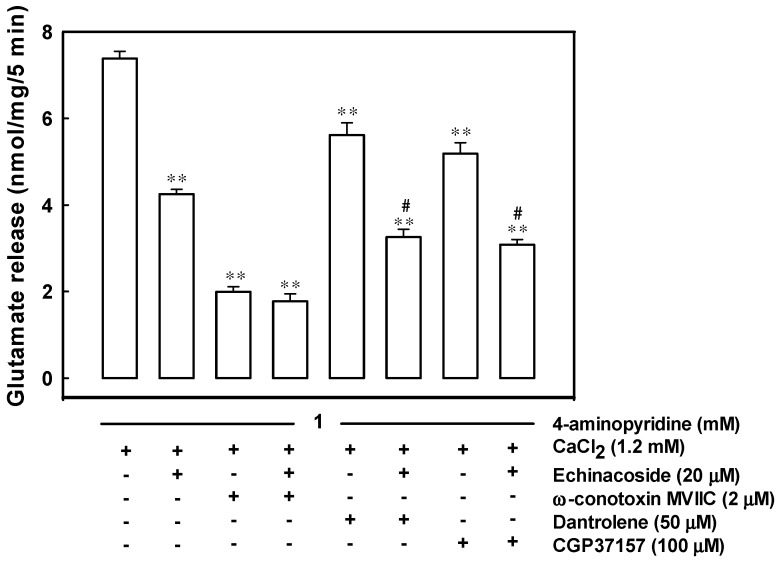
Effects of ω-conotoxin MVIIC, dantrolene, and 7-chloro-5-(2-chloropheny)-1,5-dihydro-4,1-benzothiazepin-2(3H)-one (CGP37157) on the echinacoside-mediated inhibition of 4-aminopyridine-evoked glutamate release. Glutamate release was evoked by 1 mM 4-aminopyridine in the absence (control) or presence of 20 μM echinacoside, 2 μM ω-conotoxin MVIIC, 2 μM ω-conotoxin MVIIC and 20 μM echinacoside, 10 μM dantrolene, 10 μM dantrolene and 20 μM echinacoside, 100 μM CGP37157, or 100 μM CGP37157 and 20 μM echinacoside. Echinacoside was added 10 min before depolarization and, ω-conotoxin MVIIC, dantrolene or CGP37157, 30 min prior to this. Results are mean ± S.E.M. of 5–6 independent synaptosomal preparations. **, *p* < 0.001 versus control group. #, *p* < 0.05 versus the dantrolene- or CGP37157-treated group.

Figure 2 shows that 2 μM ω-conotoxin MVIIC, an N- and P/Q-type Ca^2+^ channel blocker, reduced 4-aminopyridine-evoked glutamate release from 7.4 ± 0.2 to 2.0 ± 0.1 nmol/mg/5 min (*t*(9) = 25.35, *p* = 0.000). In the presence of ω-conotoxin MVIIC, the effect of 20 μM echinacoside on 4-aminopyridine-evoked glutamate release was nonsignificant (1.8 ± 0.2 nmol/mg/5 min; *t*(8) = 1.06, *p* = 0.32). Dantrolene (10 µM), an inhibitor of intracellular Ca^2+^ release from the endoplasmic reticulum [28], reduced 4-aminopyridine-evoked glutamate release (5.6 ± 0.3 nmol/mg/5 min; F(2,14) = 104.95, *p* = 0.000). However, in the presence of dantrolene, 20 µM echinacoside could still inhibit glutamate release significantly (3.3 ± 0.2 nmol/mg/5 min; *p* = 0.000). Similar results were observed using 100 µM 7-chloro-5-(2-chloropheny)-1,5-dihydro-4,1-benzothiazepin-2(3H)-one (CGP37157), a membrane-permeable blocker of mitochondrial Na^+^/Ca^2+^ exchange. In the five examined synaptosomal preparations, 20 µM echinacoside combined with 100 µM CGP37157 reduced 4-aminopyridine-evoked glutamate release by 48.3% ± 5.2% (F(2,13) = 136.79, *p* = 0.000), similar to the inhibition by echinacoside alone (46.2% ± 2.3%; *p* = 0.89; Figure 2).

### 2.4. Echinacoside Inhibits 4-Aminopyridine-Evoked Glutamate Release by Signaling through Protein Kinase C

**Figure 3:**
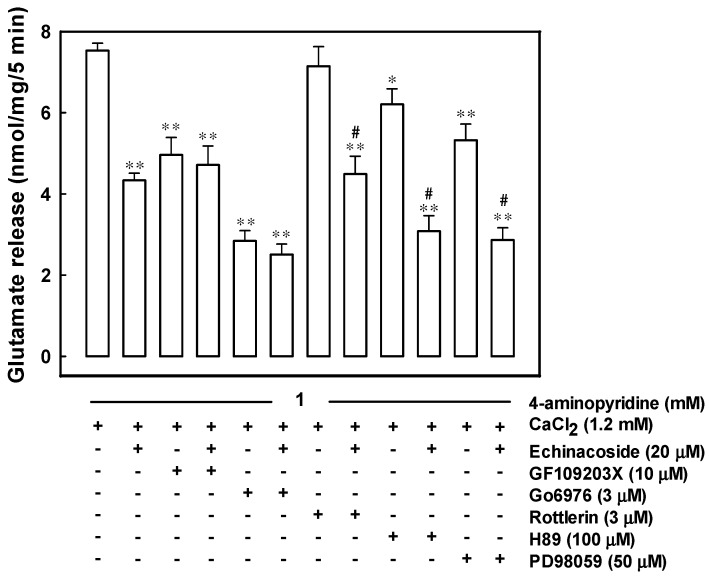
Echinacoside-mediated inhibition of 4-aminopyridine-evoked glutamate release is blocked by protein kinase C inhibitors. Glutamate release was evoked by 1 mM 4-aminopyridine in the absence (control) or presence of 20 μM echinacoside, 10 μM 2-[1-(3-dimethylaminopropyl)indol-3-yl]-3-(indol-3-yl) maleimide (GF109203X), 10 μM GF109203X and 20 μM echinacoside, 3 μM 5,6,7,13-tetrahydro-13-methyl-5-oxo-12*H*-indolo[2,3-*a*]pyrrolo[3,4-*c*]carbazole-12-propanenitrile (Go6976), 3 μM Go6976 and 20 μM echinacoside, 3 μM rottlerin, 3 μM rottlerin and 20 μM echinacoside, 100 μM N-[2-(*p*-bromocinnamylamino)ethyl]-5-isoquinolinesulfonamide (H89), 100 μM H89 and 20 μM echinacoside, 50 μM PD98059, or 50 μM PD98059 and 20 μM echinacoside. Echinacoside was added 10 min before depolarization and, GF109203X, Go697, rottlerin, H89 or PD98059, 30 min prior to this. Results are mean ± S.E.M. of 5–6 independent synaptosomal preparations. *, *p* < 0.01, **, *p* < 0.001 versus control group. #, *p* < 0.05 versus the rottlerin-, H89-, or PD98059-treated group.

As illustrated in Figure 3, 10 μM 2-[1-(3-dimethylaminopropyl)indol-3-yl]-3-(indol-3-yl) maleimide (GF109203X), a general protein kinase C inhibitor [29], reduced 4-aminopyridine-evoked glutamate release (F(2,13) = 19.46, *p* = 0.000). In the GF109203X-treated synaptosomes, 20 μM echinacoside reduced 4-aminopyridine-evoked glutamate release by only 5.5% ± 1.8% (*p* = 0.89), less than that by echinacoside alone (42.4% ± 2.3%; *p* = 0.000). Similar results were obtained with 5,6,7,13-tetrahydro-13-methyl-5-oxo-12*H*-indolo[2,3-*a*]pyrrolo[3,4-*c*]carbazole-12-propanenitrile (Go6976), a selective for Ca^2+^-dependent protein kinase C isoforms (α, βI, βII, γ) [29]. In the presence of 3 μM Go6976, 20 μM echinacoside reduced glutamate release by 11.9% ± 2.9% (*p* = 0.59), signifying a significant reduction compared with that by echinacoside alone (42.4% ± 2.3%; *p* = 0.000; Figure 3). By contrast, 3 μM rottlerin, a Ca^2+^-independent protein kinase Cδ inhibitor [30], did not significantly alter 4-aminopyridine (1 mM)-evoked glutamate release (*p* = 0.45). Nevertheless, in the presence of rottlerin, 20 μM echinacoside effectively caused an average inhibition of 37.1% ± 5.6% of the release (F(2,13) = 19.72, *p* = 0.000), similar to that by echinacoside alone (*p* = 0.41; Figure 3). In addition, the mitogen-activated protein kinase inhibitor 2-(2-amino-3-methoxyphenyl)-4H-1-benzopyran-4-one) (PD98059) (50 μM) and the protein kinase A inhibitor N-[2-(*p*-bromocinnamylamino)ethyl]-5-isoquinolinesulfonamide (H89) (100 μM) reduced 4-aminopyridine-evoked glutamate release (*p* = 0.000). Even in the presence of PD98059 or H89, 20 μM echinacoside effectively reduced the release (F(2,13) = 52.3, *p* = 0.000; Figure 3).

**Figure 4:**
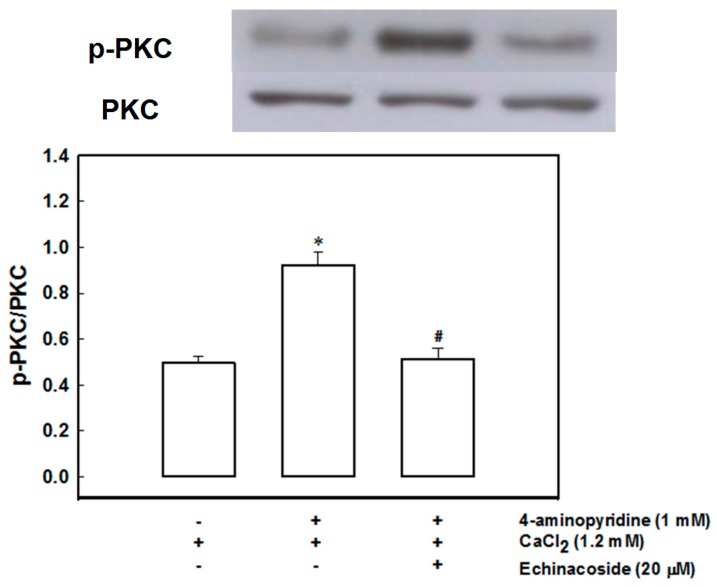
Echinacoside decreases 4-aminopyridine-induced phosphorylation of protein kinase C (PKC). Phosphorylation of PKC was detected in synaptosomal lysates by Western blotting using anti-phospho-PKC (pan) antibody. Synaptosomes were incubated for 2 min in 4-(2-hydroxyethyl)-1-piperazineethanesulfonic acid (HEPES) buffer medium that contained 1.2 mM CaCl_2_ at 37 °C in the absence (control) or presence of 1 mM 4-aminopyridine, or 1 mM 4-aminopyridine and 20 µM echinacoside added 10 min before the addition of 4-aminopyridine. Results are the mean ± S.E.M. of five independent synaptosomal preparations. *, *p* < 0.01 versus control group. #, *p* < 0.05 versus the 4-aminopyridine-treated group.

Figure 4 shows that 1 mM 4-aminopyridine increased the phosphorylation of protein kinase C in synaptosomes (*t*(4) = −6.871, *p* = 0.002). When synaptosomes were pretreated with 20 μM echinacoside for 10 min before the addition of 4-aminopyridine, 4-aminopyridine induced phosphorylation of protein kinase C considerably decreased (F(2,6) = 29.202, *p* = 0.001).

### 2.5. Echinacoside-Mediated Inhibition of Glutamate Release Does Not Involve a Gamma-Aminobutyric Acid Type A (GABA_A_) Receptor

**Figure 5:**
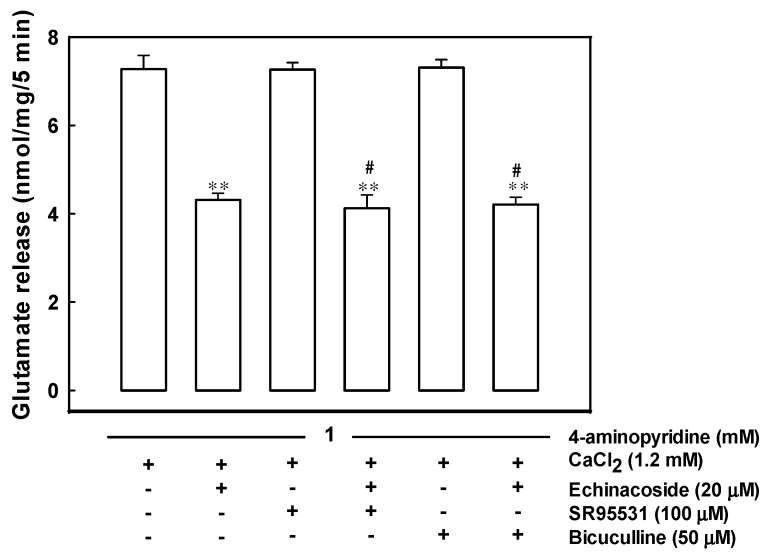
Effect of the gamma-Aminobutyric acid A (GABA_A_) receptor antagonists SR95531 and bicuculline on the echinacoside-mediated inhibition of glutamate release. Glutamate release was evoked by 1 mM 4-aminopyridine in the absence (control) or in the presence of 20 µM echinacoside, 100 µM SR95531, 100 µM SR95531 and 20 µM echinacoside, 50 µM bicuculline, or 50 µM bicuculline and 20 µM echinacoside. Results are mean ± S.E.M. of 5 independent synaptosomal preparations. **, *p* < 0.001 versus control group. #, *p* < 0.05 versus the SR95531-, or bicuculline-treated group.

In Figure 5, the effect of echinacoside on 4-aminopyridine-evoked glutamate release in the absence or presence of SR95531 (an antagonist of the GABA_A_ receptor) was compared. In addition, 100 µM SR95531 did not significantly alter 4-aminopyridine (1 mM)-evoked glutamate release. In the SR95531-treated synaptosomes, application of 20 µM echinacoside resulted in a 43% inhibition on 4-aminopyridine-evoked glutamate release (F(2,12) = 42.63, *p* = 0.000), which was not significantly different from the inhibition produced by echinacoside alone (40%; *p* = 0.000). A similar result was obtained with another GABA_A_ receptor antagonist, bicuculline (50 µM). The release measured in the presence of bicuculline and echinacoside was significantly different from that obtained in the presence of bicuculline alone (*p* = 0.000).

## 3. Discussion

In this study, echinacoside, an active compound in *Herba* *Cistanche*, inhibited 4-aminopyridine-evoked glutamate release in the rat cerebral cortex nerve terminals. The possible underlying mechanisms for the echinacoside-mediated inhibition of glutamate release are further investigated and discussed here.

### 3.1. Mechanisms Underlying Echinacoside-Mediated Inhibition of Glutamate Release

Glutamate release evoked by 4-aminopyridine comprises two components: a physiologically relevant Ca^2+^-dependent component, which is produced through exocytosis of synaptic vesicles containing glutamate; and a Ca^2+^-independent component, which originates from prolonged depolarization causing a membrane potential-mediated shift of the glutamate transporter steady-state toward the outward direction, thus affecting cytosolic glutamate efflux [31]. Here, we observed that echinacoside did not significantly inhibit 4-aminopyridine-evoked glutamate release in the presence of a Ca^2+^-free medium (Ca^2+^-independent release). Furthermore, the observed echinacoside-mediated inhibition of 4-aminopyridine-evoked glutamate release was effectively prevented by bafilomycin A1 (which depletes the glutamate content of synaptic vesicles), but not by dl-TBOA (which nonselectively inhibits all excitatory amino acid transporter subtypes). These results suggest that echinacoside affects the Ca^2+^-dependent exocytosis of glutamate release without affecting the Ca^2+^-independent cytosolic efflux of glutamate through the reversal of the nerve terminal plasma membrane glutamate transporter.

In synaptic terminals, Na^+^ channel inhibition or K^+^ channel activation stabilizes membrane excitability and consequently reduced the evoked Ca^2+^ entry and neurotransmitter release [32,33]. Therefore, the potential mechanism underlying echinacoside-mediated glutamate release inhibition involves a reduction in synaptosomal excitability. However, this possibility is untenable on the basis of two observations: (1) 4-aminopyridine-evoked membrane potential depolarization, measured with the membrane potential sensitive dye DiSC_3_(5) was unaffected by the addition of echinacoside; and (2) echinacoside did not affect the 4-aminopyridine-evoked Ca^2+^-independent glutamate release, a component of release that depends on only the membrane potential [31]. If the effect is not caused by synaptosomal excitability suppression, it may be manifested through a reduction in the activity of Ca_v_2.2 (N-type) and Ca_v_2.1 (P/Q-type) Ca^2+^ channels coupled with glutamate exocytosis in the nerve terminals [34,35,36]. By using fura-2, we demonstrate that echinacoside significantly reduces the 4-aminopyridine-evoked increase in Ca^2+^ concentration. In addition, our data show that the inhibitory effect of echinacoside on 4-aminopyridine-evoked glutamate release decreased from 42.4% ± 2.3% to 12.1% ± 3.9% after exposure to a blocker of Ca_v_2.2 (N-type) and Ca_v_2.1 (P/Q-type) Ca^2+^ channels. Furthermore, we observed that echinacoside continuted to significantly inhibit 4-aminopyridine-evoked glutamate release in the presence of intracellular Ca^2+^ release inhibitors. These results indicate that the simultaneous suppression of Ca_v_2.2 (N-type) and Ca_v_2.1 (P/Q-type) Ca^2+^ channel activity is the potentially mechanism underlying echinacoside-mediated glutamate release inhibition. However, the combined activation of Ca_v_2.2 (N-type) and Ca_v_2.1 (P/Q-type) Ca^2+^ channel activity could not block the action of echinacoside completely. Hence, other unidentified types of Ca^2+^ channels or other presynaptic pathways may be involved in the inhibition. For example, GABA_A_ receptors are present at the presynaptic level, and their activation has been shown to inhibit Ca^2+^ influx and glutamate release [37]. In the present study, the GABA_A_ receptor antagonists SR95531 and bicuculline did not block echinacoside-mediated inhibition of glutamate release, suggesting that GABA_A_ receptors are not involved in the reduction of voltage-dependent Ca^2+^ channel activity and in the subsequent inhibition of glutamate release.

Ca^2+^ entry through voltage-dependent Ca^2+^ channels activates several protein kinases associated with glutamate release in nerve terminals including mitogen-activated protein kinase, protein kinase C, and protein kinase A. Here, we demonstrate that protein kinase C inhibitors efficiently antagonized the echinacoside-mediated inhibition of glutamate release; nevertheless, the mitogen-activated protein kinase inhibitor PD98059 or the protein kinase A inhibitor H89 was ineffective. Furthermore, the 4-aminopyridine-induced phosphorylation of protein kinase C decreased in synaptosomes after pretreatment with echinacoside at a concentration effective for inhibiting glutamate release. Therefore, the signaling pathway of the echinacoside-mediated glutamate release inhibition may involve protein kinase C. Protein kinase C is an important intracellular signaling system that is present at the presynaptic level and has a crucial role in neurotransmitter exocytosis. For example, several synaptic proteins involved in the synaptic vesicle trafficking or recruitment and exocytosis, such as the myristoylated alanine-rich C kinase substrate, are phosphorylated by protein kinase C [38,39]. This phosporylation process can be increased by depolarization-stimulated Ca^2+^ entry, which facilitates glutamate release [40]. Hence, we can reasonably speculate that the inhibitory effect of echinacoside on Ca^2+^ entry observed here may reduce protein kinase C activity and consequently glutamate release.

### 3.2. Therapeutic Implications

Excitotoxicity, a pathological process caused by excessive glutamate release and glutamate receptor activation, is the major cause of neuronal death in acute and chronic brain disorders such as stroke, traumatic brain injury, Parkinson’s, and Alzheimer’s diseases [13,41], and therapeutic strategies involving glutamate release inhibition may be promising neuroprotective strategies for treating such diseases. Echinacoside has been confirmed to penetrate the blood-brain barrier (BBB) and exhibits neuroprotective effects in various in vivo models of neurotoxicity [8,10,11,12,42]. Although the mechanism of these neuroprotective effects is not completely understood, several possible mechanisms have been reported including inflammatory response inhibition, mitochondrial function stabilization, antioxidation, free radical scavenging, and neurotrophic function mimicking [5,9,12,42]. In the current study, the ability of echinacoside to reduce glutamate release from nerve terminals may also partly explain its neuroprotective mechanism. However, whether this effect contributes to the apparent therapeutic potential of echinacoside in brain disorders associated with glutamate excitotoxicity warrants further research.

## 4. Materials and Methods

### 4.1. Chemicals

Fura-2-acetoxymethyl ester (Fura-2-AM) and 3’,3’,3’-dipropylthiadicarbocyanine iodide [DiSC_3_(5)] were purchased from Invitrogen (Carlsbad, CA, USA). ω-conotoxin MVIIC, rottlerin, 2-[1-(3-dimethylaminopropyl)indol-3-yl]-3-(indol-3-yl) maleimide (GF109203X), 5,6,7,13-tetrahydro-13-methyl-5-oxo-12*H*-indolo[2,3-*a*]pyrrolo[3,4-*c*]carbazole-12-propanenitrile (Go6976) and *N*-[2-(*p*-bromocinnamylamino)ethyl]-5-isoquinolinesulfonamide (H89) were purchased from Tocris Bioscience (Bristol, UK). Echinacoside, dantrolene, dl-threo-beta-benzyl-oxyaspartate (dl-TBOA), 7-chloro-5-(2-chloropheny)-1,5-dihydro-4,1-benzothiazepin-2(3H)-one (CGP37157), 2-(2-amino-3-methoxyphenyl)-4H-1-benzopyran-4-one) (PD98059), ethylene glycol bis(β-aminoethyl ether)-*N*,*N*,*N*′,*N*′-tetraacetic acid (EGTA) and all other reagents were purchased from Sigma-Aldrich Co. (St. Louis, MO, USA).

### 4.2. Animals

Two-month old male Sprague–Dawley rats were used. Animals were housed under standardized environmental conditions (22 ± 1 °C; 50% relative humidity; 12 h light/dark cycle) and allowed unlimited access to food and water. The animals were killed by decapitation and the cerebral cortex rapidly removed at 4 °C. The experimental procedures were approved by the Fu Jen Institutional Animal Care and Utilization Committee (A10259), in accordance with the National Institutes of Health Guide for the Care and Use of Laboratory Animals. All efforts were made to minimize animal suffering and to use a minimum number of animals necessary to produce reliable results.

### 4.3. Synaptosomal Preparations

Synaptosomes were purified from the cerebral cortex of rats on discontinuous Percoll gradients as described previously [43,44]. Briefly, the tissue was homogenized in medium containing 0.32 M sucrose (pH 7.4), the homogenate was centrifuged for 10 min at 3000× *g* (5000 rpm in a JA 25.5 rotor; Beckman Coulter, Inc., Miami, FL, USA) and 4 °C, and the supernatant was centrifuged again for 12 min at 14,500× *g* (11,000 rpm in a JA 25.5 rotor). The pellet was gently resuspended in 0.32 M sucrose (pH 7.4), and an aliquot of this synaptosomal suspension (2 mL) was placed onto a 3 mL Percoll discontinuous gradient containing 0.32 M sucrose, 1 mM EDTA, 0.25 mM dl-dithiothreitol, and 3%, 10%, and 23% Percoll (pH 7.4). After centrifugation at 32,500× *g* (16,500 rpm in a JA 20.5 rotor) for 7 min at 4 °C, the synaptosomes were recovered from between the 10% and the 23% Percoll bands, and they were diluted in a final volume of 30 mL of HEPES buffer medium (140 mM NaCl, 5 mM KCl, 5 mM NaHCO_3_, 1 mM MgCl_2_·6H_2_O, 1.2 mM Na_2_HPO_4_, 10 mM glucose, and 10 mM HEPES (pH 7.4)). Following further centrifugation at 27,000× *g* (15,000 rpm in a JA 25.5) for 10 min, the synaptosome pellet was resuspended in 3 mL of HEPES buffer medium, and the protein content was determined using a Bradford assay. Finally, 0.5 mg of the synaptosomes suspension was diluted in 10 ml of HEPES buffer medium and centrifuged at 3000× *g* (5000 rpm in a JA 20.1 rotor) for 10 min. The supernatant was discarded, and the pellets containing the synaptosomes were stored on ice and used within 4–6 h.

### 4.4. Glutamate Release

Glutamate release was assayed by on-line fluorimetry as described previously [45,46]. Synaptosomal pellets were resuspended in HEPES buffer medium (0.5 mg/mL) and preincubated at 37 °C for 10 min in the presence of 16 µM bovine serum albumin to bind any free fatty acids released from synaptosomes during preincubation. A 2-mL aliquot of the synaptosomes was transferred to a stirred cuvette containing 2 mM NADP^+^, 50 units of glutamate dehydrogenase, and 1.2 mM CaCl_2_, and the fluorescence of NADPH was measured in a Perkin-Elmer LS-55 spectrofluorimeter (PerkinElmer Life and Analytical Sciences, Waltham, MA, USA) at excitation and emission wavelengths of 340 and 460 nm, respectively. As synaptosomes are not amenable to electrical stimulation, the potassium channel blocker 4-aminopyridine was used to stimulate glutamate release. 4-aminopyridine destabilizes the membrane potential and is thought to cause repetitive spontaneous Na^+^ channel-dependent depolarization that closely approximates in vivo depolarization of the synaptic terminal that leads to the activation of voltage-dependent Ca^2+^ channels and neurotransmitter release [47]. Data were obtained at 2 s intervals. A standard of exogenous glutamate (5 nmol) was added at the end of each experiment. The value of the fluorescence change produced by the standard addition was used to calculate the released glutamate as nanomoles of glutamate per milligram of synaptosomal protein (nmol/mg). Release values quoted in the text are levels attained at steady-state after 5 min of depolarization (nmol/mg/5 min). Cumulative data were analyzed using Lotus 1-2-3 spreadsheets (IBM, White Plains, NY, USA) and MicroCal Origin (OriginLab Corporation, Northampton, MA, USA).

### 4.5. Plasma Membrane Potential

The plasma membrane potential was determined with a membrane-potential-sensitive dye, DiSC_3_(5) [48]. Synaptosomes were resuspended in HEPES buffer medium, and 2 mL aliquots were transferred to a stirred cuvette containing 5 µM DiSC_3_(5) at 37 °C in a Perkin–Elmer LS-55 spectrofluorometer (PerkinElmer Life and Analytical Sciences, Waltham, MA, USA). After allowing the mixture to equilibrate for 3 min, the fluorescence was determined at excitation and emission wavelengths of 646 and 674 nm, respectively. Data were collected at 2 s intervals. Cumulative data were analyzed using MicroCal Origin (OriginLab Corporation, Northampton, MA, USA) and expressed in fluorescence units.

### 4.6. Cytosolic Ca^2+^ Concentration ([Ca^2+^]_C_)

The [Ca^2+^]_C_ was measured with the Ca^2+^ indicator fura-2. Synaptosomes (0.5 mg/mL) were preincubated in HEPES buffer medium containing 5 µM fura-2 and 0.1 mM CaCl_2_, for 30 min at 37 °C in a stirred test tube. After fura-2 loading, synaptosomes were centrifuged in a microcentrifuge for 30 s at 3000× *g* (5000 rpm). The synaptosomal pellets were resuspended in HEPES buffer medium, and the synaptosomal suspension was stirred in a thermostatted cuvette in a Perkin-Elmer LS-55 spectrofluorometer (PerkinElmer Life and Analytical Sciences, Waltham, MA, USA). CaCl_2_ (1 mM) was added after 3 min and further additions were made after an additional 10 min. Fluorescence data were accumulated at excitation wavelengths of 340 and 380 nm (emission wavelength 505 nm) at 2 s intervals. [Ca^2+^]_C_ (nM) was calculated using calibration procedures [49] and equations described previously [50]. Cumulative data were analyzed using MicroCal Origin (OriginLab Corporation, Northampton, MA, USA).

### 4.7. Western Blotting

Synaptosomes were homogenized in a lysis buffer (10 mM HEPES buffer, pH 7.4), 1% Triton X-100, and protease inhibitor mixture. Lysates were clarified by centrifugation, and protein concentration was determined using a protein assay kit (Bio-Rad Laboratories, Hercules, CA, USA). Equal amounts of proteins were separated by sodium dodecyl sulphate-polyacrylamide gel electrophoresis (SDS-PAGE) and transferred to nitrocellulose membrane. The membranes were blocked with Tris-buffered saline that contained 5% low-fat milk and incubated with appropriate primary antibody (phospho-protein kinase C (pan), 1:3000, NOVUS Biologicals Inc., Beverly, MA, USA) overnight at 4 °C. After three washes in Tris-buffered saline, the membrane was then treated with the secondary horseradish peroxidase-conjugated antibody (1:3000) for 1 h at room temperature. The membranes were then washed at least three times with Tris-buffered saline and visualized using the enhanced chemiluminescence system (Amersham, Buckinghamshire, UK). An aliquot of samples was loaded and probed with anti-PKC antibody for detection of PKC as a loading control. The level of expression or phosphorylation was assessed by band density, which was quantified by densitometry. Densitometric quantification of bands was analyzed using Syngene software (Synoptics, Cambridge, UK).

### 4.8. Statistical Analysis

Data were obtained from a single synaptosomal preparation and were not independent of one another. To test the significance of the effect of a drug versus control, a two-tailed Student’s *t*-test was used. When an additional comparison was required (such as whether a second treatment influenced the action of echinacoside), a one-way ANOVA followed by Tukey’s test was used. Analysis was completed via software SPSS (17.0; SPSS Inc., Chicago, IL, USA). Data are expressed as mean ± S.E.M.; significance was evaluated at *p* < 0.05 for all statistical measures.

## 5. Conclusions

This is the first study demonstrating that echinacoside inhibits glutamate release from rat cerebrocortical synaptosomes by reducing Ca^2+^ influx through Cav2.2 and Cav2.1 channels, and this release inhibition is likely dependent on the suppression of the protein kinase C pathway, at least in part. The present finding is valuable because it provides a novel insight into the mechanisms of action of echinacoside in the brain.

## Figures and Tables

**Table 1:** Effect of echinacoside on membrane potential and cytosolic Ca^2+^ levels from rat cortical synaptosomes.

	Membrane Potential (Fluorescence Units)		Cytosolic [Ca^2+^] (nM)	
	Basal	4-Aminopyridine (1 mM)	Basal	4-Aminopyridine (1 mM)
Control	5.6 ± 0.3	14.8 ± 0.4	144.6 ± 3.1	219.3 ± 6.9
Echinacoside	5.2 ± 0.3	14.8 ± 0.4	143.7 ± 3.5	176.5 ± 6.7 **

Each value is mean ± S.E.M. of independent experiments using synaptosomal preparations from 5–6 animals. **, *p* < 0.001 versus the control group.
